# Structural Predictors of Lung Function Decline in Young Smokers with Normal Spirometry

**DOI:** 10.1164/rccm.202307-1203OC

**Published:** 2024-01-04

**Authors:** Andrew I. Ritchie, Gavin C. Donaldson, Eric A. Hoffman, James P. Allinson, Chloe I. Bloom, Charlotte E. Bolton, Gourab Choudhury, Sarah E. Gerard, Junfeng Guo, Luana Alves-Moreira, Lorcan McGarvey, Elizabeth Sapey, Robert A. Stockley, K. P. Yip, Dave Singh, Tom Wilkinson, Malin Fageras, Kristoffer Ostridge, Olaf Jöns, Enrica Bucchioni, Chris H. Compton, Paul Jones, Karen Mezzi, Jørgen Vestbo, Peter M. A. Calverley, Jadwiga A. Wedzicha, on behalf of the British Early COPD Network (BEACON) Cohort Investigators, Sophie Kempka

**Affiliations:** ^1^ National Heart and Lung Institute, Imperial College London, London, United Kingdom;; ^2^ AstraZeneca, Cambridge, United Kingdom;; ^3^ Department of Radiology and; ^4^ Roy J. Carver Department of Biomedical Engineering, Medicine and Biomedical Engineering, University of Iowa, Iowa City, Iowa;; ^5^ Royal Brompton Hospital, London, United Kingdom;; ^6^ NIHR Nottingham Biomedical Research Centre,; ^7^ Centre for Respiratory Research, NIHR Nottingham, Translational Medical Sciences, School of Medicine, University of Nottingham, Nottingham, UK;; ^8^ ELEGI and COLT Laboratories, Queen’s Medical Research Institute, Edinburgh, United Kingdom;; ^9^ Wellcome-Wolfson Institute for Experimental Medicine, School of Medicine, Dentistry and Biomedical Sciences, Queen’s University Belfast, Belfast, United Kingdom;; ^10^ Belfast Health and Social Care Trust, Belfast, United Kingdom;; ^11^ Institute of Inflammation and Ageing, University of Birmingham, Birmingham, United Kingdom;; ^12^ Division of Infection, Immunity and Respiratory Medicine, University of Manchester, Manchester, United Kingdom;; ^13^ Faculty of Medicine, University of Southampton, Southampton, United Kingdom;; ^14^ National Institute for Health and Care Research Southampton Biomedical Research Centre, University Hospital Southampton, Southampton, United Kingdom;; ^15^ AstraZeneca, Gothenburg, Sweden;; ^16^ Boehringer Ingelheim International GmbH, Ingelheim am Rhein, Germany;; ^17^ Chiesi Farmaceutici S.p.A., Parma, Italy;; ^18^ GlaxoSmithKline, Brentford, United Kingdom;; ^19^ Novartis Pharma AG, Basel, Switzerland; and; ^20^ Institute of Life Course and Medical Sciences, University of Liverpool, Liverpool, United Kingdom

**Keywords:** chronic obstructive pulmonary disease, early COPD, FEV_1_, lung function, quantitative computed tomography

## Abstract

**Rationale:**

Chronic obstructive pulmonary disease (COPD) due to tobacco smoking commonly presents when extensive lung damage has occurred.

**Objectives:**

We hypothesized that structural change would be detected early in the natural history of COPD and would relate to loss of lung function with time.

**Methods:**

We recruited 431 current smokers (median age, 39 yr; 16 pack-years smoked) and recorded symptoms using the COPD Assessment Test (CAT), spirometry, and quantitative thoracic computed tomography (QCT) scans at study entry. These scan results were compared with those from 67 never-smoking control subjects. Three hundred sixty-eight participants were followed every six months with measurement of postbronchodilator spirometry for a median of 32 months. The rate of FEV_1_ decline, adjusted for current smoking status, age, and sex, was related to the initial QCT appearances and symptoms, measured using the CAT.

**Measurements and Main Results:**

There were no material differences in demography or subjective CT appearances between the young smokers and control subjects, but 55.7% of the former had CAT scores greater than 10, and 24.2% reported chronic bronchitis. QCT assessments of disease probability–defined functional small airway disease, ground-glass opacification, bronchovascular prominence, and ratio of small blood vessel volume to total pulmonary vessel volume were increased compared with control subjects and were all associated with a faster FEV_1_ decline, as was a higher CAT score.

**Conclusions:**

Radiological abnormalities on CT are already established in young smokers with normal lung function and are associated with FEV_1_ loss independently of the impact of symptoms. Structural abnormalities are present early in the natural history of COPD and are markers of disease progression.

Clinical trial registered with www.clinicaltrials.gov (NCT 03480347).

**At a Glance Commentary**

**Scientific Knowledge on the Subject**

Understanding the root causes of chronic obstructive pulmonary disease (COPD) and developing effective treatments are crucial for COPD research. Most patients are identified when significant structural damage is present, with airspace enlargement, airway thickening, and reduced numbers of small airways. Existing treatment can ameliorate the resulting symptoms but does not reverse the underlying pathophysiological problem, making earlier disease identification a priority.

**What This Study Adds to the Field**

This large prospective cohort study showed that radiological abnormalities measured on quantitative computed tomography relate to smoking history and reflect an already established degree of subclinical small airway dysfunction. Radiological abnormality and, separately, symptom scores related to an increased decline in FEV_1_. Greater decline was seen in those who self-reported chronic bronchitis. This finding was confirmed by several sensitivity analyses. Our data have identified abnormalities early in the natural history of the disease, which can be monitored to assess physiological and pathological impact. More important, we have shown that even a relatively short period of regular tobacco smoking results in identifiable lung abnormalities before smokers typically receive diagnoses of COPD.

Chronic obstructive pulmonary disease (COPD) is currently characterized by the presence of respiratory symptoms and structural changes in airways and lungs leading to airflow obstruction that is persistent and often progressive (1). Most patients are identified when significant structural damage is present with airspace enlargement, airway thickening, and reduced numbers of small airways (2, 3). Existing treatment can ameliorate the resulting symptoms (4) but does not reverse the underlying pathophysiological problem, making earlier disease identification a priority (5).

Our understanding of the natural history of COPD changed with the recognition of early-life loss of lung function in many cases (6). A large proportion of patients with COPD achieve their expected lung growth and experience an accelerated decline in FEV_1_ thereafter, usually due to tobacco smoking (6, 7). Several life-spanning birth cohorts have shown that smoking causes respiratory symptoms to emerge gradually and is associated with accelerated loss of lung function (8, 9). New quantitative computed tomography (CT) imaging techniques allow a more detailed analysis of lung structure *in vivo* (10, 11). To date, such studies have focused on those with well-established COPD or older individuals in whom COPD has not developed despite heavy smoking (12, 13). Studying such groups is unlikely to identify the earliest stages of COPD development (5).

We hypothesized that even modest tobacco exposure in midlife would lead to structural change and symptoms in susceptible individuals and that these would be associated with an accelerated decline in lung function. To address this, we established a multicenter prospective observational study in the United Kingdom, recruiting smokers (the BEACON [British Early COPD Network] cohort) 30–45 years of age with normal findings on spirometry who underwent quantitative thoracic CT (QCT) imaging, symptom scoring, and detailed physiological measurements over a median of 32 months of follow-up. In addition, we compared our baseline data with those from a group of healthy nonsmokers with similar demographic characteristics.

## Methods

### Study Design and Participants

**Figure 1.**
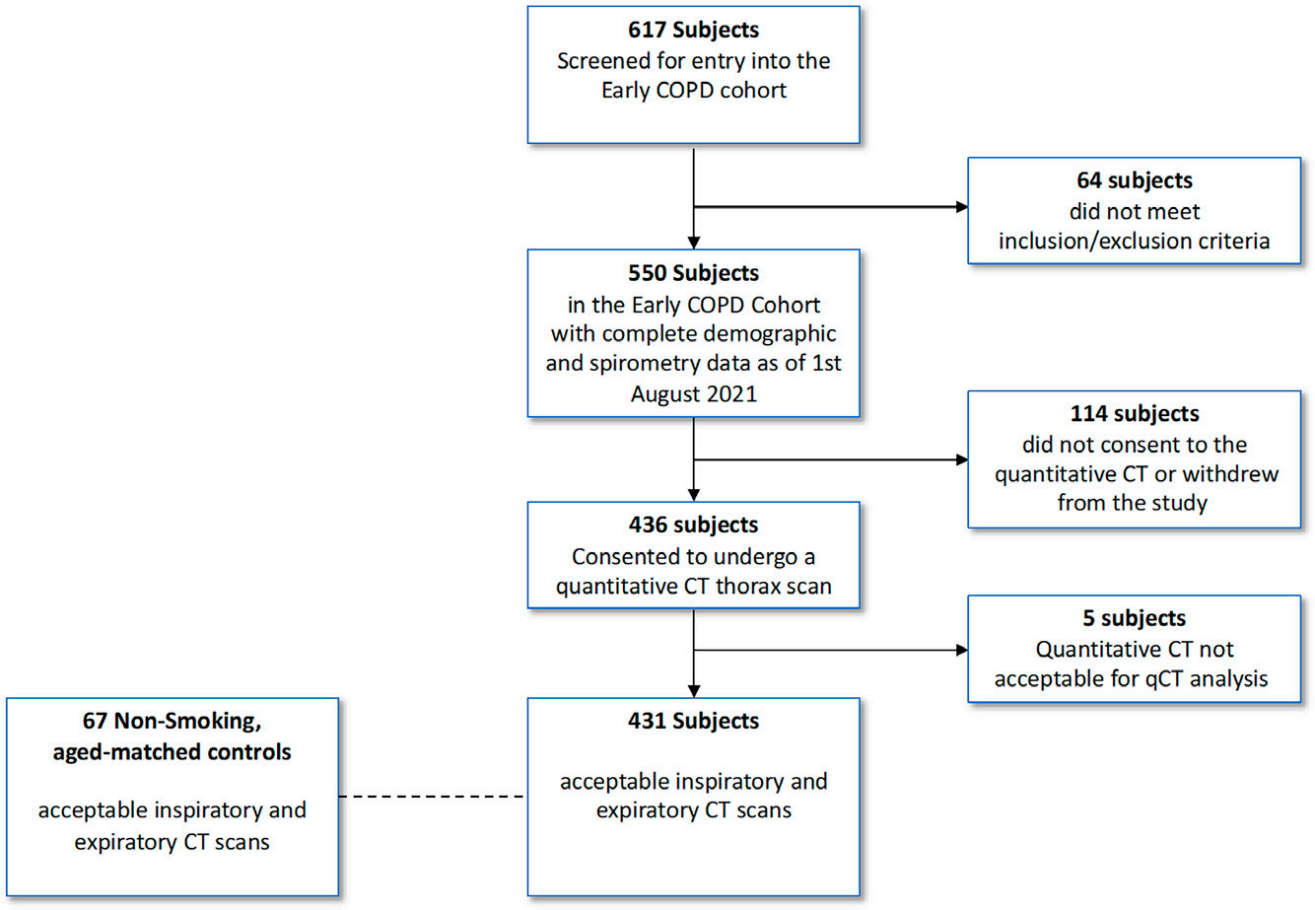
Flow diagram outlining selection of the study cohort and healthy control subjects. COPD = chronic obstructive pulmonary disease; CT = computed tomography; qCT = quantitative computed tomography.

The BEACON cohort was recruited from seven academic centers (*see* Tables E1 and E2 in the online supplement) between February 2018 and February 2020. It enrolled 30- to 45-year-old current tobacco smokers (defined as having regularly consumed tobacco within the preceding 7 d) with >10 pack-year smoking histories, postbronchodilator FEV_1_ of at least 80% predicted, and body mass index < 35 kg/m^2^. Any history of chronic lung, cardiovascular, diabetic, or autoimmune disease, a current cancer diagnosis, or regular cannabis use led to exclusion (*see* Table E3; Figure 1). All participants were offered smoking cessation support. At enrollment, demographics, symptoms, and physiological data were recorded (*see* the online supplement and Table E4). Six-monthly follow-up visits including lung function assessment were scheduled (*see* the online supplement and Table E4) but were delayed if within 6 weeks of a respiratory infection. Participants underwent quantitative inspiratory- and expiratory-phase CT scans (coached TLC and coached residual volume) within 180 days of recruitment.

Healthy never-smokers (lifetime smoking history < 100 cigarettes) aged 30–50 years without histories of chronic lung disease and with preserved spirometry (FEV_1_ > 80% predicted and FEV_1_:FVC ratio > 0.7) were recruited by the University of Iowa between November 2017 and September 2018 (Figure 1).

All participants gave written informed consent, and the study protocols were approved by the relevant research ethics committees (*see* the online supplement) and registered at ClinicalTrials.gov (NCT 03480347).

### QCT Scanning

The imaging protocol for both cohorts was standardized as detailed by Sieren and colleagues (*see* the online supplement) (14) and analyzed centrally (VIDA Diagnostics, Inc.). The disease probability measure (DPM) approach (15, 16) was used to provide quantitative classification of whole-lung nonemphysematous gas trapping (DPM-defined air trapping [DPM_AirTrap_], indicating functional small airway disease [fSAD]), emphysema (DPM-defined emphysema [DPM_Emph_]), and lung volume meeting healthy criteria for each participant (*see* Table E5). DPM quantifies the voxel-to-voxel difference in Hounsfield units between matched inspiratory and expiratory images to estimate the probability of air trapping such that the probability is inversely proportional to the relative differences in Hounsfield units. When quantified using DPM analysis, air trapping reflects what has been referred to previously as fSAD (17). The upper limit of normal for emphysema has been previously defined (18).

On the inspiratory scans, we also estimated an index of airway wall thickness (Pi10) as the square root of the wall area of an airway with a 10-mm inner perimeter. Larger Pi10 values indicate thicker airway walls relative to luminal size (19). Interstitial components of the lung were characterized using three-dimensional texture analysis (adaptive multiple-feature method [AMFM]) (20–22). The AMFM is a machine-learning approach that classifies regional parenchymal textures such as ground-glass opacities (GGOs) and bronchovascular bundles (*see* the online supplement). Briefly, the AMFM-based texture analysis quantifies GGOs, or other lung tissue characteristics, as a percentage of total lung volume at TLC by using grayscale patterns within CT images. Intrapulmonary vasculature was investigated by assessing the ratio of small vessel volume (SVV_0.75_; vessels with radii <0.75 mm) to total pulmonary vessel volume (TPVV). Total lung and TPVV segmentations were generated using convolutional neural networks (23). The size of each vessel was calculated by first identifying the vessel center line and then calculating the distance from each center line voxel to the nearest boundary voxel.

### Other Variables

Spirometry and body plethysmography lung volume measurements were obtained within the BEACON cohort at each visit according to the American Thoracic Society and European Respiratory Society guidelines (24, 25), and predicted values were estimated using the Global Lung Function Initiative reference equations (26). Respiratory symptoms were assessed using the COPD Assessment Test (CAT) questionnaire. Chronic bronchitis was self-reported as a productive cough for at least three months in two consecutive years. Smoking burden was measured in pack-years.

### Statistical Analysis

Categorical data are summarized as frequencies and percentages. For continuous data, normally distributed variables are summarized as mean (SD) and never-normally distributed variables as median (interquartile range [IQR]). We compared the baseline demographics and spirometric values of BEACON participants and never-smoking control subjects using two-sample *t* tests for continuous variables and chi-square statistics for categorical variables. The imaging data were highly skewed, so we tested for between-group differences using Mann-Whitney tests.

For the longitudinal analysis of the follow-up measurements made every six months during follow-up (median 32 mo) in the BEACON cohort, we used random-effects linear regression to investigate the association between radiological abnormalities and change in FEV_1_ over time. To examine the correlations between CT abnormalities and lung function, we chose lung function parameters that were expressed as percentage predicted or ratios to minimize issues with sex and body size (*see* Figure E4). We adjusted for age and sex at initial recruitment and smoking status at each follow-up visit (model 1). We also investigated the association between baseline symptoms (measured using the CAT score) and change in FEV_1_ over follow-up, using random-effects linear regression (model 2). As chronic bronchitis is known to be associated with accelerated FEV_1_ decline, the presence of self-reported chronic bronchitis (*see* the online supplement) was explored in two sensitivity analyses. The first sensitivity analysis used random-effects linear regression to assess the interaction with FEV_1_ change when adjusted for age, sex, and smoking status. The second sensitivity analysis added chronic bronchitis as a covariate to statistical models 1 and 2.

Follow-up lung function measurements were not available in the never-smoking control group. *P* values were two sided, with Bonferroni adjustment for multiple comparisons (with adjustment for the 12 comparisons across the two groups), and values less than 0.05 were considered to indicate statistical significance for all analyses. All statistical analyses were conducted using Stata version 17 (StataCorp) or Prism version 9.2 (GraphPad Software).

## Results

### Study Sample

Of 550 individuals in the BEACON cohort, 431 (78%) (all current smokers, 41% women), with a median age of 38 (IQR, 35–42) years and a mean FEV_1_ of 101% predicted (SD, 11.6%), underwent CT scanning (Figure 1). The CT scans of five participants were technically unacceptable and excluded from analysis. One hundred fourteen cohort members either declined consent for scanning or withdrew. Of the remaining 431 participants (median smoking history, 16 pack-years [IQR, 12–21 pack-years]; median CAT score, 10 [IQR, 6–15]), 24.2% reported symptoms of chronic bronchitis, and none took regular inhaled pharmacological treatment. The mean postbronchodilator FEV_1_ was 101% predicted, and the plethysmographic lung volumes were normal. The smoking and never-smoking groups were similar in age, sex, body mass index, and spirometric values (Table 1, Figure 1). Three hundred sixty-eight smokers provided prospective longitudinal spirometric data, with a median follow-up time of 32 months (IQR, 24.2–41.5 mo).

**Table 1.** Demographics and Clinical Characteristics of Study Participants

	BEACON Cohort	Healthy, Never-Smoking Control Subjects	*P* Value
Total number	431	67	—
Age, yr, median (IQR)	39 (35–42)	36 (31–46)	0.621
Sex, *n* (%)			
Male	251 (60.0)	35 (52.2)	0.284*
Female	167 (40.0)	32 (47.8)	—
BMI, kg/m^2^, mean (SD)	25.8 (4.09)	25.8 (3.99)	0.870
Ethnicity, *n* (%)			
Asian	10 (2.3)	4 (4.4)	—
Black	66 (15.3)	2 (3.0)	—
Hispanic	0 (0)	6 (9.0)	—
White	355 (82.4)	54 (80.6)	—
Tobacco smoking history			
Smoking pack-years, median (IQR)	16 (12–21)	—	—
Age started regularly smoking, yr, median (IQR)	15 (14–17)	—	—
Cumulative years smoking, median (IQR)	22 (19–26)	—	—
Chronic bronchitis symptoms, *n* (%)^†^	101 (24.2)	—	—
LRTI and/or CAP in previous year, *n*/yr, mean (SD)	0.45 (0.28)	—	—
CAT score	10 (6-15)	—	—
mMRC grade, *n* (%)			
0	291 (69.6)	—	—
1	120 (28.7)	—	—
2	7 (1.7)	—	—
Follow-up time, mo, median (IQR)	32 (24.2–41.5)	—	—
Quantitative computed tomography			
LAA at −950 Hounsfield units inspiratory, %, mean (SD)	0.92 (1.1)	1.52 (1.1)	<0.001
*n*/total *N* (%) with >5% emphysema	4 (1.0)	0 (0)	0.600^‡^
LAA at −856 Hounsfield units expiratory, %, mean (SD)	2.3 (6.2)	0.5 (1.0)	<0.001
*n*/total *N* (%) with >20% gas trapping	8 (1.9)	0 (0)	1.00^‡^
Prebronchodilator spirometry			
FEV_1_, L, mean (SD)			
Male	4.09 (0.69)	4.23 (0.52)	0.690
Female	3.03 (0.49)	3.32 (0.48)	0.450
FEV_1_, % predicted, mean (SD)	94.1 (11.4)	99.8 (10.4)	0.344
FVC, L, mean (SD)			
Male	5.31 (0.90)	5.24 (0.68)	0.472
Female	3.86 (0.69)	3.97 (0.50)	0.440
FEV_1_:FVC ratio, mean (SD)	0.78 (0.08)	0.82 (0.05)	0.288
Postbronchodilator spirometry			
FEV_1_, L, mean (SD)			
Male	4.28 (0.70)	—	—
Female	3.15 (0.47)	—	—
FEV_1_, % predicted, mean (SD)	101.8 (11.9)	—	—
FVC, L, mean (SD)			
Male	5.36 (0.90)	—	—
Female	3.89 (0.60)	—	—
FEV_1_:FVC ratio, mean (SD)	0.80 (0.06)	—	—
FEV_1_:FVC ratio < 0.7, *n* (%)	18 (4.1)	—	—
Bronchodilator reversibility, mean (SD)			
FEV_1_, ml	154.2 (173.5)	—	—
FEV_1_, %	7.8 (26.8)	—	—
FVC, ml	36.7 (178.0)	—	—
Plethysmography			
TLC, L, mean (SD)			
Male	7.23 (1.13)	—	—
Female	5.35 (0.79)	—	—
TLC, %, mean (SD)	100.0 (12.2)	—	—
RV, L, mean (SD)			
Male	1.88 (0.44)	—	—
Female	1.52 (0.39)	—	—
RV, %, mean (SD)	116.8 (24.4)	—	—
RV:TLC ratio, %, mean (SD)			
Male	26.0 (5.5)	—	—
Female	28.3 (4.4)	—	—

*Definition of abbreviations*: BEACON = British Early COPD Network; BMI = body mass index; CAP = community-acquired pneumonia; CAT = COPD Assessment Tool; IQR = interquartile range; LAA = low-attenuation area; LRTI = lower respiratory tract infection; mMRC = Modified Medical Research Council Dyspnea Scale; RV = residual volume.

* Chi-square test.

^†^ Chronic bronchitis was defined as a productive cough for at least three months in two consecutive years.

^‡^ Fisher exact test.

### Radiological Abnormalities among Smokers Relative to Never-Smokers

**Figure 2.**
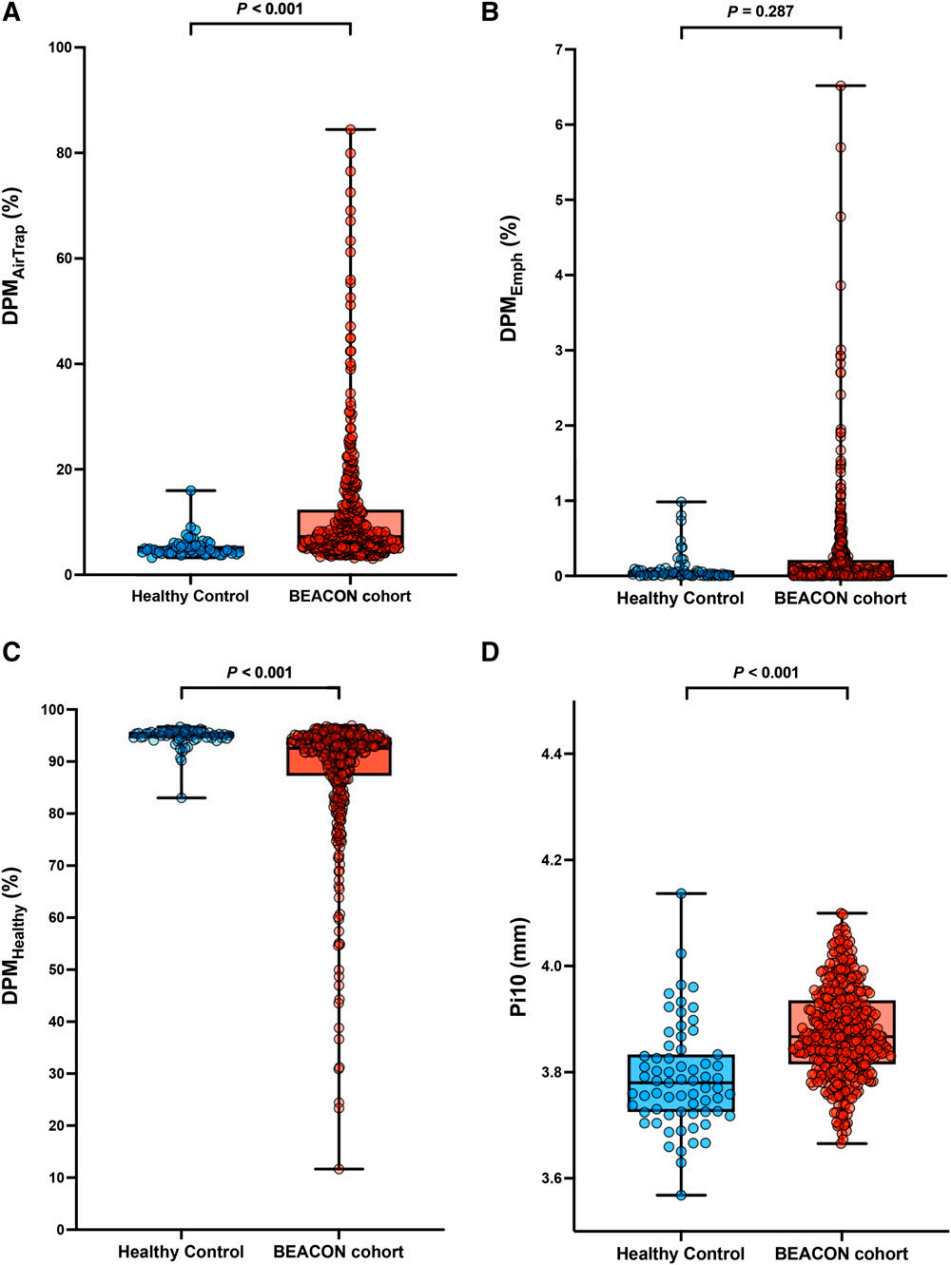

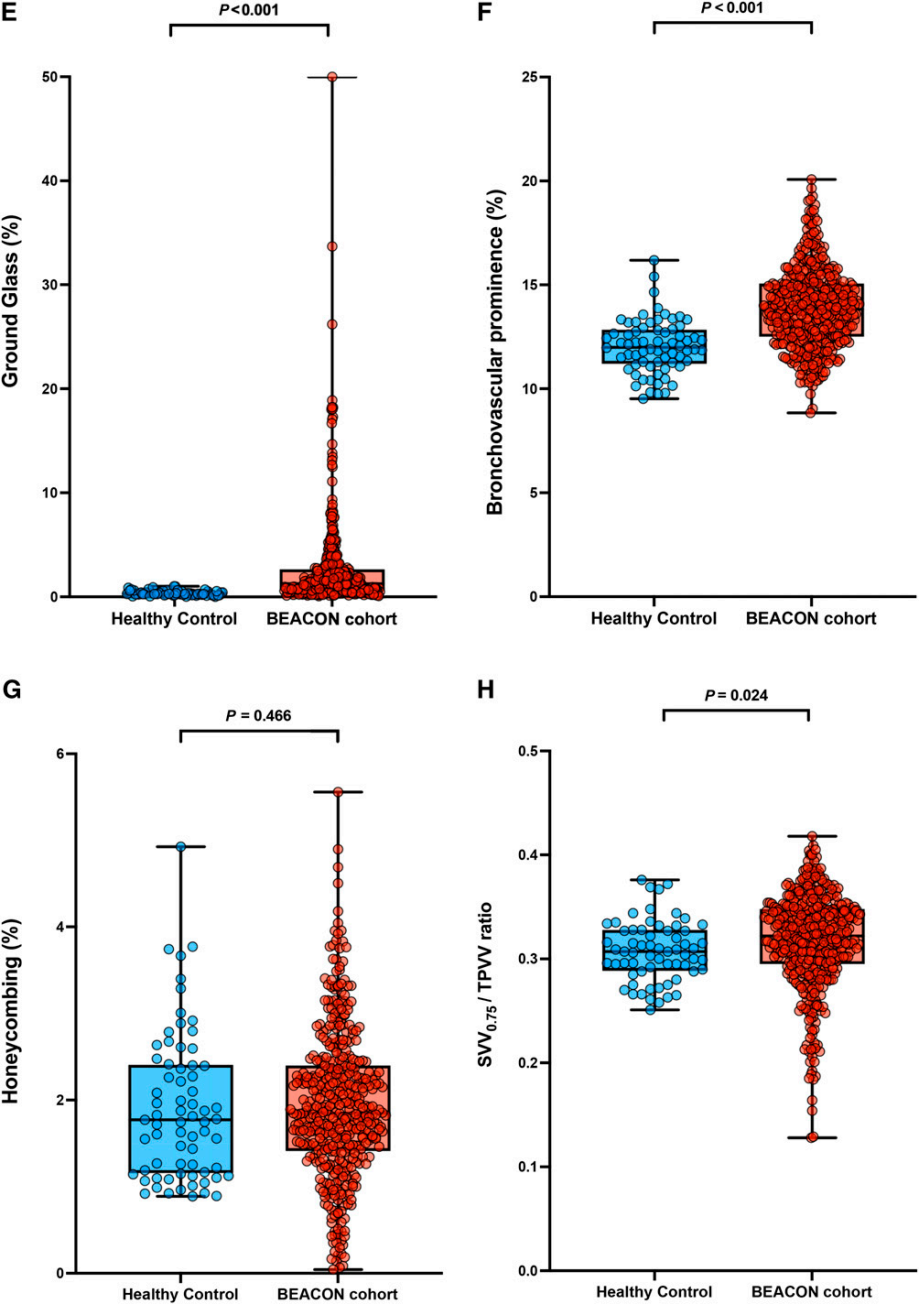
Comparison of quantitative computed tomography parameters by disease probability measure and three-dimensional texture analysis methods between the BEACON cohort and healthy control subjects. (*A–D*) Differences in DPM_AirTrap_ (*A*), DPM_Emph_ (*B*), DPM_Healthy_ (*C*), and Pi10 (*D*). (*E–G*) Difference in prominent bronchovascular bundles (expressed as a percentage of lung field) (*E*), ground-glass opacity (*F*), and honeycombing (*G*), using the three-dimensional texture analysis method. (*H*) Difference in the ratio of SVV_0.75_ to TPVV. Comparisons were made using the Mann-Whitney test. BEACON = British Early COPD Network; DPM_AirTrap_ = disease probability measure–defined air trapping; DPM_Emph_ = disease probability measure–defined emphysema; DPM_Healthy_ = disease probability measure–defined healthy lung; Pi10 = square root of the wall area of a hypothetical airway with a 10-mm inner perimeter (larger values indicate thicker airway walls); SVV_0.75_ = small vessel volume (vessels with radii ⩽0.75 mm); TPVV = total pulmonary vessel volume.

DPM analysis demonstrated that the BEACON cohort participants had a higher percentage of DPM_AirTrap_ compared with never-smoking control subjects (7.4% [IQR, 5.3–12.4%] vs. 4.7% [IQR, 4.2–5.5%], respectively; *P* < 0.001) and a greater Pi10 (3.85 [IQR, 3.81–3.88] vs. 3.78 [IQR, 3.73–3.83], respectively; *P* < 0.001) (Figure 2). There was no significant difference in DPM_Emph_ (0.05% [IQR, 0.01–0.20%] vs. 0.03% [IQR, 0.01–0.07%]; Mann-Whitney *P* = 0.146). The BEACON cohort participants had lower percentages of DPM-defined normal lung compared with never-smokers (92.5% [IQR, 87.4–94.7%] vs. 95.3% [IQR, 94.5–95.5%]; *P* < 0.001) and a higher ratio of SVV_0.75_ to TPVV (0.32 [IQR, 0.29–0.35] vs. 0.30 [IQR, 0.29–0.33]; *P* < 0.001).

**Figure 3.**
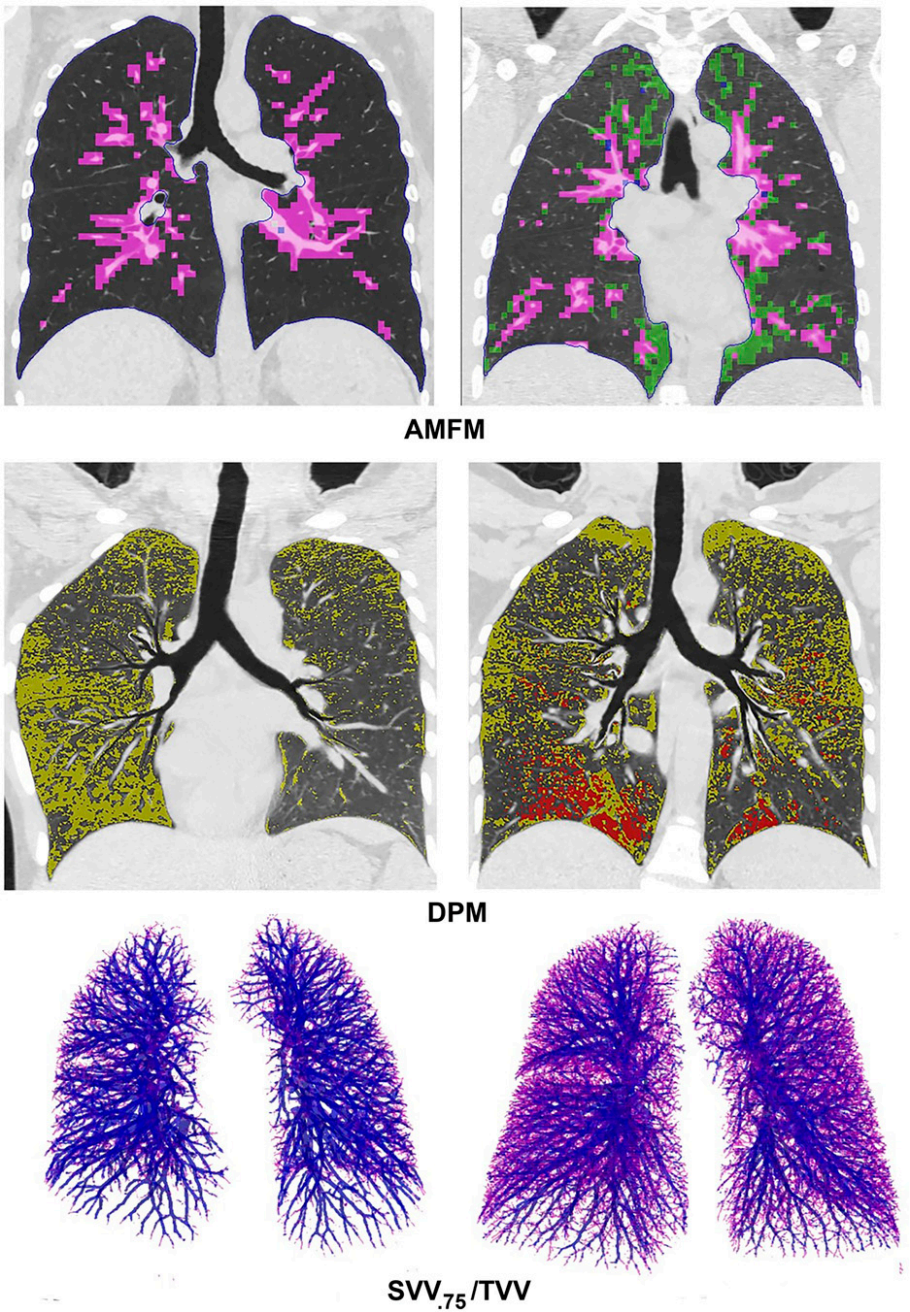
Visual examples of the image-based metrics. Top panels demonstrate midcoronal sections from a nonsmoking subject (right) with 9% AMFM-defined bronchovascular bundles (pink) and 0.6% ground-glass opacities (green) and a participant in the smoking cohort with 15% bronchovascular bundles and 18% ground-glass opacities. Middle panels demonstrate two members of the baseline smoking cohort. On the left is a participant with disease probability measure (DPM)–defined 25% air trapping (yellow) but no emphysema, and on the right is a participant with DPM-defined 29% air trapping (yellow) and 5.7% emphysema (red). The DPM results are shown within a midcoronal section of a topographic multiplanar reformatted (VIDA Diagnostics) (37) view in which the airways are flattened into a single plane with the parenchyma similarly warped. In the bottom panels, smoking participants at the extremes of the SVV_.75_/TVV distribution are displayed, with ratios of 0.085% (left) and 0.404% (right). Vessels with diameters of 0.75 mm or less are displayed in red, and larger vessels are displayed in dark purple. AMFM = adaptive multiple-feature method; SVV_.75_ = small vessel volume (vessels with radii ⩽0.75 mm); TVV = total vessel volume.

AMFM texture analysis revealed that the BEACON cohort had an increased percentage of prominent bronchovascular bundles compared with never-smokers (13.8% [IQR, 12.5–15.1%] vs. 12.0% [IQR, 11.2–12.9%], respectively; *P* < 0.001) and GGO (1.30% [IQR, 0.68–2.63%] vs. 0.30% [IQR, 0.22–0.46%], respectively; *P* < 0.001). Both groups had minimal AMFM-defined honeycombing of the lung parenchyma (1.8% [IQR, 1.4–2.4%] vs. 1.8% [IQR, 1.2–2.4%], respectively; *P* = 0.466) (Figure 2). This AMFM honeycomb texture can alternatively be related to an enhanced “CT density gradient” at the borders of vascular bundles, possibly associated with inflammatory processes (27). Figure 3 summarizes visual differences between these two groups.

QCT abnormalities increased with age and tobacco consumption independently of age, but there was substantial collinearity between tobacco pack-years and age at recruitment. (*see* Figures E1–E3). There was no significant gender difference in the occurrence of QCT abnormality.

### Associations among CT Measures, Lung Function, and Symptoms in Smokers

Although some of the associations between FEV_1_ and FEV_1_:FVC ratio achieved nominal statistical significance, generally the relationships were weak. There were significant associations between DPM_AirTrap,_ Pi10, and ground-glass opacification and the ratio of residual volume to TLC (*see* Figure E4). The median CAT score in the BEACON cohort participants was 10.5 (IQR, 6–15). There were no significant differences in QCT measurements between those reporting high (>10) and low (⩽10) CAT scores (*see* Figure E5).

### Rate of FEV_1_ Decline and Baseline Radiological Abnormalities and Symptoms in Younger Adult Smokers

The annualized FEV_1_ decline rate in the BEACON cohort was −36.4 ml/yr (95% confidence interval [CI], −44.4 to −28.4 ml/yr; *P* < 0.001). When adjusting for age, sex, and smoking, we found that increases in six pathological QCT measurements were associated with accelerated annualized FEV_1_ decline (Table 2). Increased DPM_AirTrap_ was associated with an additional −1.5 ml/yr per 1% increase in the CT measurement (95% CI, −2.1 to −0.1 ml/yr; *P* < 0.001), while DPM_Emph_ was associated with an additional −19.5 ml/yr decline per 1% increase (95% CI, −33.5 to −5.6 ml/yr; *P* < 0.001). For a 0.1-mm increase in the Pi10 measurement, there is an additional −9.1 ml/yr (95% CI, −33.5 to −7.1 ml/yr; *P* < 0.001) loss of FEV_1_. An increase in the volume of small blood vessels (as assessed by the ratio of SVV_0.75_ to TPVV), was significantly associated with faster FEV_1_ decline (an additional −1.1 ml/yr per 0.01 increment in the ratio [95% CI, −1.3 to −0.8 ml/yr]; *P* < 0.001). Increased ground-glass opacification (−3.4 ml/yr per 1% increase [95% CI, −5.5 to −1.3 ml/yr]; *P* < 0.001) and percentage of prominent bronchovascular bundles (−2.6 ml/yr per 1% increase [95% CI, −3.2 to −2.0 ml/yr]; *P* < 0.001) also related to accelerated FEV_1_ decline. To explore the effect of baseline lung function on the relationship between QCT measurements and FEV_1_ decline, we added the recruitment FEV_1_ value to our models as a covariate. This made little difference to the magnitude or direction of the relationships between CT and FEV_1_ decline (*see* Table E7).

**Table 2.** Examining the Association between Computed Tomography Measurements and Symptom Burden on Baseline FEV_1_ and FEV_1_ Longitudinal Change in the Study Cohort

	Difference in Baseline FEV_1_ (*ml*) (95% CI)	*P* Value	Annualized Rate of FEV_1_ Change (*ml/yr*) (95% CI)	*P* Value
Model 1: CT measurements	Per 1% increase in CT parameter			
Air trapping, emphysema, and medium-large airway wall thickness				
DPM_AirTrap_, %	−1.1 (−4.1 to 6.1)	NS	−1.5 (−2.1 to −0.1)	<0.001
DPM_Emph_, %	92.3 (26.4 to 184.3)	NS	−19.5 (−33.5 to −5.6)	0.036
Pi10 (per 0.1-mm increase)	−79.7 (−1,136 to 976.8)	NS	−9.1 (−11.3 to −7.1)	<0.001
Vascular measurement				
SVV_0.75_:TPVV (per 0.01 increase in ratio)	17.7 (51 to 300)	0.036	−1.1 (−1.3 to −0.8)	<0.001
Additional structural measurements				
Ground-glass opacity, %	−10.4 (−25.3 to 4.5)	NS	−3.4 (−5.5 to −1.3)	0.006
Bronchovascular prominence, %	−26.7 (−57.2 to 0.4)	NS	−2.6 (−3.2 to -2.0)	<0.001
Model 2: symptoms	Per 1-unit increase in CAT score			
CAT score (per additional 1 unit)	−13.4 (−22.3 to −4.5)	0.003	−1.4 (−2.7 to −0.2)	0.025

*Definition of abbreviations*: CAT = COPD Assessment Test; CI = confidence interval; CT = computed tomography; DPM_AirTrap_ = disease probability measure–defined air trapping; DPM_Emph_ = disease probability measure–defined emphysema; NS = nonsignificant; Pi10 = measurement of bronchial wall thickness; SVV_0.75_ = small vessel volume (vessels with radii ⩽0.75 mm); TPVV = total pulmonary vessel volume.

Separate generalized least squares random-effects models are presented for each CT parameter. Models were adjusted for FEV_1_ decline interaction with time, smoking status (e.g., quit), gender, and age. All *P* values are adjusted for multiple comparisons.

Self-reported chronic bronchitis was associated with an additional −32.6 ml/yr (95% CI, −48.3 to −17.0 ml/yr; *P* < 0.001) loss of FEV_1_. When including chronic bronchitis as a covariate in the model assessing the association of radiological abnormality and FEV_1_ change, DPM_Emph_ was the only parameter for which chronic bronchitis augmented FEV_1_ decline (−28.6 ml/yr [95% CI, −45.3 to −12.0 ml/yr]; *P* = 0.006) (*see* Table E7).

The median CAT score was 10 (IQR, 6–15) (Table 1). An increase in baseline CAT score by 1 unit was associated with a lower baseline FEV_1_ (−13.2 ml/unit [95% CI, −22.2 to −4.2 ml/unit]; *P* = 0.004) (Table 2) and with accelerated FEV_1_ decline (an additional 3.0 ml/yr per unit [95% CI, −3.6 to −2.3 ml/yr per unit]; *P* < 0.001) (Table 2).

One hundred three smokers (28%) showed annual FEV_1_ declines of more than 60 ml/yr (*see* Table E8). Those with this accelerated decline were more commonly male (67% vs. 56%; *P* = 0.01) and more symptomatic at baseline (median CAT score, 12.1 vs. 10.5; *P* = 0.03) despite similar tobacco consumption. There was no difference in radiological abnormality scores between these groups.

Not all subjects enrolled and scanned then attended follow-up to provide longitudinal lung function, but this group did not significantly differ from those who remained (*see* Table E9).

### Smoking Status Change during Follow-Up

All participants were active smokers at enrollment. Change in smoking status was included in the longitudinal modeling because 58 participants (15.8%) intermittently quit smoking at one or more follow-up visits. A total of six (1.6%) participants quit tobacco throughout the study observation period.

## Discussion

Our data show for the first time the extent to which smokers younger than 45 years with normal findings on spirometry have changes in their small airways, lung parenchyma, and pulmonary vasculature. These abnormalities are unrelated to their symptomatology but increase with greater tobacco exposure. In addition, these changes predict a faster rate of lung function loss but are not the only mechanism for this process, as an increased symptom score also independently predicts functional decline.

Prior studies quantitatively analyzing QCT scan–derived metrics suggested that fSAD and emphysema can develop before measurable change in larger airways (12, 13). However, those studies focused on older individuals with already established COPD (12, 16). Our study provides evidence that smokers commonly begin to develop small airway dysfunction and emphysema at a much younger age than previously reported (15). Although this observational study was not designed to test causality, the relationship found between the presence of radiological abnormalities and smoking, in particular pack-years accrued, would fit with smoking’s being the primary cause of these radiological abnormalities.

FEV_1_ decline has remained the main marker of COPD progression, and it is central to current concepts of COPD development during adulthood (28). Our FEV_1_ decline rate of 36.4 ml/yr compares with similar data (8, 28), as do the effect of chronic bronchitis and the association we find between baseline CAT score and accelerated FEV_1_ decline (8, 29). We also describe for the first time a relationship between a number of QCT measurements and subsequent rate of FEV_1_ decline.

Increased fSAD, emphysema, and bronchial wall thickening (Pi10) are associated with an increase in the decline of subsequently measured FEV_1_. The overall burden of these radiological abnormalities found in the BEACON cohort conforms to expectations compared with other studies of COPD in older patients (15). Overall, the quantity of emphysema measured in our participants was low, but we found that a 1% increase in this measurement was related to a ∼10-fold increase in FEV_1_ loss compared with fSAD and Pi10. Relatively novel measurements of interest, such as the increase in ratio of SVV_0.75_ to TPPV, GGOs, and prominence of bronchovascular bundles were associated with a subsequently accelerated rate of FEV_1_ decline. The higher proportion of small vessels in the BEACON cohort is potentially explained by downstream increased resistance in inflamed lung regions (30). An increased regional pulmonary parenchymal perfusion heterogeneity has been shown to be reduced by a single dose of oral sildenafil, supporting the hypothesis that the increased resistance is associated with regional hypoxic vasoconstriction in the inflamed regions (31, 32). An alternative explanation is that the greater enlargement of the SVV_0.75_ relative to the TPVV is because the increased size of the small vessels brings them within the resolution of the scanner, and thus more small vessels enter into the calculation as opposed to enlargement of existing vessels.

Not all smokers showed each of these changes. The was a modest association between DPM_AirTrap_ and DPM_Emph_, with a similar association between DPM_Emph_ and pulmonary vessel volume. The parenchymal changes were strongly related to each other, but the airway wall thickness was not related to other changes, emphasizing the heterogeneity of lung damage present. The lack of a clear association between the CT changes and baseline physiology is unsurprising given the study entry criteria but shows that these changes are yet to significantly affect lung mechanics and patient symptomatology, thereby representing a truly early phase of the process. Tobacco burden expressed as pack-years was related the extent of small airways damage as seen in older smokers (33). Age *per se* was not responsible for the absence of this significant abnormality in our control group. Although our subjects reported a range of CAT scores, and 25% had symptoms of chronic bronchitis, there were no differences in QCT appearance between symptomatic and asymptomatic individuals.

A study strength is the comparison between our younger smokers and nonsmokers who followed an identical scanning protocol. The prebronchodilator lung function of BEACON subjects was lower than that of control subjects, a finding noted among older “healthy” smokers (34). Although the geographical differences between these two groups are a possible limitation, it is uncertain whether this would lead to a material difference in results. Multiple lung function trajectories can lead to symptomatic COPD, and we chose to study one clearly defined closely associated with tobacco smoking and classically described by Fletcher and Peto (35). It is possible that a different pattern of radiological damage would be seen in individuals with suboptimal lung growth. Understanding the root causes of COPD and developing effective interventions are a priority for COPD research (5). Not all subjects enrolled then provided follow-up, but these participants did not significantly differ from those who remained. Additional analyses, such as the degree of airway mucus plugging (36) and total airway count (3), would shed further light on the lung structure in our cohort, while interval CT scanning would help understand damage progression and the occurrence of new abnormalities. Nevertheless, these limitations do not detract from our finding that radiological abnormalities, classically associated with established COPD, are prevalent among young adult smokers.

### Conclusions

Our study shows that these radiological abnormalities relate to prior smoking history, reflect an already established degree of subclinical small airway dysfunction, and provide novel QCT data that relates to subsequent FEV_1_ decline. Understanding the root causes of COPD and developing effective treatments are priorities for COPD research (5, 28). Our data have identified abnormalities early in the natural history of the disease, which can be monitored to assess physiological and pathological impact. More important, we have shown that even a relatively short period of regular tobacco smoking results in identifiable lung abnormalities before smokers typically receive diagnoses of COPD.
